# Invasive forest pathogens in Europe: Cross-country variation in public awareness but consistency in policy acceptability

**DOI:** 10.1007/s13280-018-1046-7

**Published:** 2018-03-23

**Authors:** Louise Eriksson, Johanna Boberg, Thomas L. Cech, Tamara Corcobado, Marie-Laure Desprez-Loustau, Ari M. Hietala, Marília Horta Jung, Thomas Jung, Hatice Tuğba Doğmuş Lehtijarvi, Funda Oskay, Slavtcho Slavov, Halvor Solheim, Jan Stenlid, Jonàs Oliva

**Affiliations:** 1 Department of Geography and Economic History, Umeå University, 901 87 Umeå, Sweden; 2 Department of Psychology, Umeå University, Umeå, Sweden; 3 Department of Crop and Forest Sciences, ETSEA, University of Lleida, Av. Alcalde Rovira Roure, 191, 25198 Lleida, Spain; 4 Department of Forest Mycology and Plant Pathology, Swedish University of Agricultural Sciences, Uppsala, Sweden; 5 Phytophthora Research Centre, Faculty of Forestry and Wood Technology, Mendel University in Brno, Zemědělská 3, 613 00 Brno, Czech Republic; 6 Department of Forest Protection, Phytopathology, Federal Research and Training Centre for Forests, Natural Hazards and Landscape (BFW), Vienna, Austria; 7 UMR1202 BIOGECO, INRA, University Bordeaux, 33610 Cestas, France; 8 Norwegian Institute of Bioeconomy Research, PO Box 115, 1431 Ås, Norway; 9 Laboratory of Molecular Biotechnology and Phytopathology, Centre for Mediterranean Bioresources and Food, University of Algarve, Algarve, Portugal; 10 Department of Botany, Faculty of Forestry, Süleyman Demirel University, 32600, Isparta, Turkey; 11 Department of Forest Entomology and Protection, Faculty of Forestry, Çankırı Karatekin University, 18200 Çankırı, Turkey; 12 Biotic Stress Group, AgroBioInstitute, Agricultural Academy, 8 Dragan Tzankov Blvd., 1164 Sofia, Bulgaria; 13 Department of Forest Mycology and Plant Pathology, Swedish University of Agricultural Sciences, Box 7026, 750 07 Uppsala, Sweden; 14 Department of Forest Protection, Phytopathology, Federal Research and Training Centre for Forests, Natural Hazards and Landscape (BFW), Seckendorff-Gudent-Weg 8, 1131 Vienna, Austria

**Keywords:** Media, Multilevel models, Problem awareness, Risk experience, Tree diseases

## Abstract

Political action can reduce introductions of diseases caused by invasive forest pathogens (IPs) and public support is important for effective prevention. The public’s awareness of IP problems and the acceptability of policies aiming to combat these pathogens were surveyed in nine European countries (*N* = 3469). Although awareness of specific diseases (e.g., ash dieback) varied, problem awareness and policy acceptability were similar across countries. The public was positive towards policies for informational measures and stricter standards for plant production, but less positive towards restricting public access to protected areas. Multilevel models, including individual and country level variables, revealed that media exposure was positively associated with awareness of IP problems, and strengthened the link between problem awareness and policy acceptability. Results suggest that learning about IPs through the media and recognizing the associated problems increase policy acceptability. Overall, the study elaborates on the anthropogenic dimension of diseases caused by IPs.

## Introduction

Globalization facilitates the introduction of animals, plants, pathogens, and microbes to areas beyond their natural range. Introduced species may become invasive, i.e., establish reproducing populations in these new areas, spread and, in some cases (especially for pathogens), cause negative impact on resident species. Similar to invasive plants and animals, forest pathogen invasions into Europe have increased exponentially over the last 30 years (Desprez-Loustau 2009; Hulme et al. 2009; Roques et al. 2009). The introductions of diseases caused by invasive forest pathogens (IP) can have detrimental effects on several ecosystem functions and services, including extraction of raw materials from the forest (e.g., timber), and aesthetics when trees in recreational areas are affected (Santini et al. 2013; Lovett et al. 2016). Invasions by IPs have a strong anthropogenic dimension, as the IPs are mainly spread via the trade of living plants, the transport of packaging materials, and human recreational activities (Cushman and Meentemeyer 2008; Santini et al. 2013; Jung et al. 2016; Lovett et al. 2016; Potter and Urquhart 2017). The vast majority of IP introductions are unintentional, e.g., the IP propagules (spores, mycelia) hitch hike within imported plant material (Liebhold et al. 2012). Microbes with harmless relationships with their co-evolved hosts may behave as severe pathogens when in contact with evolutionary naive hosts in introduced areas (Stenlid and Oliva 2016). Examples of previous devastating pandemics by invasive forest pathogens causing the mortality of keystone forest tree species include Dutch elm disease and Chestnut blight in North America and Europe (Brasier 2000; Elliott and Swank 2008). Since it is difficult to eliminate an introduced species once it has been established, it is imperative to take action to prevent their introduction.

With a shared economic market and regulatory framework, the European Union illustrates well the challenges faced when balancing between economic development and environmental protection (e.g., combining free trade and movement of products with protection against IPs). For effective prevention and management of IPs, the appropriate legislation and policy require public support (Marzano et al. 2015; Klapwijk et al. 2016). In contrast to environmental threats such as oil spills and deforestation, IPs, similar to climate change, operate on a much longer time scale, with delayed environmental and economic consequences. Hence, the public is being asked to change their behavior and accept policies related to something that is not necessarily noticeable yet. Nevertheless, studies from the UK reveal that even though awareness of IPs is low, the public is concerned and support certain management methods, such as sanitary cuttings, felling affected trees and/or biological control applications, whereas clear cutting healthy forest areas or the use of genetic modifying techniques (GM) remain controversial (Fuller et al. 2016; Jepson and Arakelyan 2017). In addition, there is evidence that the public is to some extent willing to contribute themselves (e.g., avoid bringing plants from abroad) (Urquhart et al. 2017). Limited data are available about public perceptions of IPs in different countries, and the public’s willingness to accept policies aiming to prevent the introduction and spread of these pathogens (Marzano et al. 2015). The aim of the present study was to examine public awareness and acceptability of policy measures aiming to combat IPs in nine European countries. There is a need to understand how public support is built up on an individual level, including the roles of personal encounters with IPs in gardens or forests, and indirect learning via other sources such as the mass media, in different biophysical and socio-economic contexts.

## Theoretical framework

### Risk experience, problem awareness, and policy acceptability

On an individual level, direct personal experience of a risk is often believed to result in a contextualized experience and a stronger willingness to respond. However, the role of direct versus indirect experience has been debated. In relation to climate change, for example, the nature and context of the experience of flooding (a potential consequence of climate change) and the interaction with indirect experiences (through the media) are likely important for the response (Reser et al. 2014). Whereas risk research suggests that both direct and indirect risk experiences may instigate appraisals of the risk (Reser and Swim 2011), value theories, such as the value-belief-norm theory, propose that internal values and beliefs may be even more important in determining the perceived seriousness of a risk (Stern 2000). Hence, in line with value theories, problem awareness of IPs should be influenced not only by risk experience but also by what people value in the forest (e.g., forest biodiversity or production). In turn, people aware of a problem tend to show higher levels of acceptability of policies aiming to combat the problem (Stern 2000), indicating that awareness of tree diseases and IPs should be associated with acceptability of policies directed at IPs. While studies have confirmed a link between reasons for valuing forests and acceptability of tree health management (Fuller et al. 2016), the roles of direct and indirect risk experiences and problem awareness for acceptability of policy directed at IPs have not been examined yet.

### External conditions

Public opinion is not generated in a vacuum but formed over time in a biophysical and socio-economic context (Qin and Flint 2010). Responses to forest threats have for example been linked to biophysical vulnerability and community characteristics (e.g., socioeconomics), highlighting the need to consider external conditions when analyzing public opinion on risks (Qin and Flint 2010; Flint et al. 2012). According to the social amplification of risk framework (Kasperson et al. 1988), risk events are communicated to the public via risk signals (e.g., images), which interact with psychological and societal processes. The transference of information about the risk runs through social amplification stations, such as scientists informing about the risk and the media, which in turn are expected to lead to behavioral responses in the society. The media contribute to the intensification or attenuation of the risk signals and may be particularly important for how the public perceives intangible risks, including IPs (Tomlinson 2016; Fellenor et al. 2017). External conditions such as media reports may influence public opinion directly or act as moderators. For example, the congruence between attitudes and behaviors and between the individual’s value and belief system may depend on how supportive the context is (Steg et al. 2014). Thus, a more supportive context may make attitudes more aligned with values and beliefs. Even though there is reason to believe that external conditions influence public opinion on IPs, these relationships have not been examined yet.

### Aims of the study

Risk experience, in terms of seeing or hearing about the risk, is expected to influence risk responses such as policy acceptability (Reser and Swim 2011), but evidence of how experience of tree diseases and IPs influence policy acceptability is lacking. Using a survey in nine countries covering different parts of Europe, we examined the relationships between direct and indirect experience of tree diseases and IPs, awareness, problem awareness, and the acceptability of policy measures aiming to combat IPs at the European scale. The study focused on eight specific IPs highly relevant in a European context and the policy measures included regulatory measures directed at plant production and trade, regulation of the public’s access to protected areas, and informational measures. We analyzed the importance of individual (e.g., experience of IPs), and country-level variables (e.g., media attention) for awareness of IP problems and policy acceptability. Based on risk research and value theory (Stern 2000; Reser and Swim 2011), we expected the public’s acceptability of policies to combat IPs to be correlated with risk experience (in terms of seeing or hearing about IPs) and awareness of problems associated with IPs and tree diseases in general. We furthermore expected that external conditions such as share of forest, production level, number of IPs, and media attention in each country may be related to public opinion. Since a supporting context may align attitudes with internal beliefs and values (Steg et al. 2014), we furthermore expected that awareness of IP problems and policy acceptability should be more strongly related when problems associated with IPs are highlighted in the media (i.e., a cross-level interaction).

## Materials and methods

### Sample and procedure

An online survey was conducted in January 2016 in Austria, Bulgaria, France, Norway, Portugal, Spain, Sweden, Turkey, and the UK by the market research company Multiscope. Participants were selected from the company’s online panel using a quota selection procedure to ensure representativeness of the samples. With the exception of Bulgaria and Turkey, the panel members and, hence, the samples were representative for their respective populations regarding age and gender. When survey responses from approx. 385 participants per country had been received, the survey was closed (*N* = 3469).

Gender and age distributions were comparable for the samples from Austria, France, Norway, Portugal, Spain, Sweden, and the UK: between 49 and 52% of the respondents in each country were female and approximately 30, 25, 25, and 20% of the respondents were aged 18–34 years, 35–49 years, 50–64 years, or older than 64 years, respectively. Between 51 and 75% of the respondents in the different countries lived in urban settings (> 10 000 inhabitants); between 9 and 20% had received an education in natural sciences/agriculture, and between 7 and 26% had a forest owner in the household. The Bulgarian and Turkish samples deviated from their respective populations and from the other country samples, with fewer older respondents, more urban respondents, a higher proportion of respondents with an education in natural sciences/agriculture, and a higher proportion of respondents that had a forest owner in the household. Results for Bulgaria and Turkey should therefore be interpreted with caution.

### Measures

The questionnaire was developed in English and subsequently translated to the main language in each country. To ensure that questions entailed similar meaning, the questions were back-translated and queries discussed. Questions were asked about the respondents’ gender, age group, place of residence, whether the respondent had received a formal education in biology, ecology, forestry, agriculture, or gardening (i.e., natural sciences/agriculture), and whether the respondent was a member of a forest-owning household. Subsequently, awareness, experience, problem awareness, and policy acceptability were assessed (see Table 1). Respondents were asked whether they had heard of eight diseases caused by IPs (see Table 2), indicating an *awareness of IPs* (yes/no). Direct experience was assessed on a scale of 1–5 in terms of whether the respondent had seen diseased trees nearby (*direct experience of tree disease*). Furthermore, dummy variables were created based on whether or not the respondent had come into contact with any of the eight diseases through personal observations (*direct experience of IPs*) or via the media (*indirect experience of IPs*) (only asked if they had stated that they were aware of the pathogen). Problem awareness was assessed more generally in relation to tree diseases as a broad concept, and specifically by focusing on IPs. Local and national awareness of tree disease problems were assessed on a scale of 1–5 (*local and national tree disease problems*). A measure of the respondent’s *awareness of IP problems* on a scale of 1–5 was based on three items reflecting the expected impact of IPs on biodiversity, recreational experiences, and economic forest values (including a “don’t know” option). After removing “don’t know” responses, the means of the items were used to create an index variable with high internal reliability (*α* = 0.80) (e.g., DeVellis 2012).

**Table 1:** Overview of measures in the survey

Concept	Measure	Response scale
Awareness of IPs	Have you heard of the following tree diseases caused by nonnative pathogens?	
	Dutch elm disease, ash dieback, Phytophthora decline on oak, beech or chestnut, alder Phytophthora, oak powdery mildew, chestnut blight, pine wood nematode, and pine pitch canker.	Yes/no
Direct experience of tree diseases	Trees in my neighborhood or in the countryside nearby look sick/diseased (loss of leaves, wilting, yellowing, etc.)	1–5 (totally disagree, completely agree)
Direct experience of IPs	Personal observations in: 1) Public parks and gardens, 2) Woodlands/forests or recreational areas, 3) Own garden, 4) Own woodland/forest.	Multiple answers possible
Indirect experience of IPs	Having heard or read about them in: 1) Mass media (e.g., radio or TV news, newspapers), 2) Specialized magazines, programs or webpages.	Multiple answers possible
Local awareness of tree disease problems [local tree disease problems]	Tree diseases threaten natural settings nearby (e.g., parks and countryside)	1–5 (totally disagree, completely agree)
National awareness of tree disease problems [national tree disease problems]	Tree diseases threaten trees and woodlands/forests in [the respective country]	1–5 (totally disagree, completely agree)
Awareness of IP problems [IP problems]	To what extent do you believe that tree diseases caused by nonnative pathogens could lead to the following consequences in [the respective country]?	
	A reduction in the number of native species in woodlands/forests (loss of biodiversity), a detrimental impact on recreational experiences in woodlands/forests, and a reduction in the economic value of woodlands/forests	1–5 (not at all, to a great extent), don’t know
Policy acceptability	A range of different measures could be used to prevent the introduction and spread of nonnative tree pathogens in [the respective country]. These measures may involve both pros and cons to the environment, you, and your country. Please indicate whether you are in favor or against their implementation.	
	An increase in tree disease and tree health education (e.g., information campaigns aimed at the general public) [education]	1–5 (completely against, neither in favor nor against, completely in favor)
	Introduction of more stringent health standards for plant production within Europe [stringent standards for plant production]	
	Introduction of a labeling system to inform end-consumers of the country of origin of living plant material (flowers, ornamental plants and trees for planting in the garden) [labeling of plant origin]	
	Reduction in the import of living plants (flowers, ornamental plants and trees for planting in the garden) from countries outside Europe [reduction in import of living plants]	
	Reduction in the import of timber products from countries outside Europe [reduction in import of timber]	
	Reduction in public access to protected areas (nature reserves, national parks) in order to prevent the introduction of new forest pathogens (e.g., through soil or dirt on boots) [reduction in public access to protected areas]	
	No action

**Table 2:** Public awareness of specific diseases caused by IPs (standardized within country) and awareness of at least one IP

	Turkey	Bulgaria	Spain	Portugal	France	Austria	UK	Sweden	Norway
Dutch elm disease (*Ophiostoma ulmi*)	**1.47**	− 1.09	0.17	− 1.21	− 0.74	− 1.00	**2.09**	**2.15**	**2.15**
Ash dieback (*Hymenoscyphus fraxineus*)	− 0.26	1.06	− 1.69	− 0.59	0.52	0.17	0.78	− 0.55	− 0.07
Phytophthora decline on oak, beech or chestnut (*Phytophthora sp.*)	0.03	0.01	0.04	**1.28**	0.36	0.16	− 0.53	− 0.36	− 0.47
Alder Phytophthora (*Phytophthora alni* s.l.)	− 1.84	− 1.09	− 1.34	− 1.39	− 1.62	-0.55	− 0.85	− 0.83	− 0.64
Oak powdery mildew (*Erysiphe alphitoides*)	1.05	**1.75**	0.61	0.05	0.55	**2.08**	− 0.21	0.82	0.67
Chestnut blight (*Cryphonectria parasitica*)	0.03	− 0.15	0.32	1.13	0.90	0.54	0.09	− 0.20	− 0.03
Pine wood nematode (*Bursaphelenchus xylophilus*)	0.03	− 0.62	**1.09**	0.17	− 1.05	− 0.84	− 0.77	− 0.49	− 0.74
Pine pitch canker (*Fusarium circinatum*)	− 0.51	0.13	0.80	0.55	**1.09**	− 0.57	− 0.61	− 0.55	− 0.87
Awareness of at least one IP (%)	70.4	78.3	57.0	68.4	59.4	66.3	84.9	68.3	43.6

*Note*. The scientific name of the causative pathogen is enclosed within brackets. The highest level of awareness in each country is in bold

*Policy acceptability* was assessed on a scale of 1–5 in terms of whether the respondents favored or opposed the seven policy measures (i.e., an attitude) (Eagly and Chaiken 1993): a reduction in the import of living plants and a reduction in the import of timber products from countries outside Europe, a reduction in public access to protected areas, the introduction of a labeling system to inform end-consumers about the country of origin of living plant material, an increase in tree disease and tree health education, the introduction of more stringent health standards for plant production, and no action. The item ‘no action’ was reversed before creating an index variable of policy acceptability using the means of the policy measures. The internal reliability was good (*α* = 0.74).

### Country variables

Four country variables were created to reflect external conditions potentially important for public opinion. *Share of forest* was created based on the percentage of forest and other woodland (FOREST EUROPE 2015), and an indicator of the importance of forest for *production* was formed based on the amount of roundwood and sawnwood production in 2013 (the most recent year with complete data for the countries examined, Eurostat 2016). Based on Santini et al. (2013), we created a variable reflecting to what extent IPs can be considered a problem in terms of *the number of IPs*. However, data for this variable were only available for Austria, France, Norway, Spain, Sweden, and the UK. Furthermore, we conducted a Google search of the eight IPs investigated in this study using the same disease names as in the online survey, in the main language of each country within quotation marks. Searches were restricted to each country, but no time period or other restriction was used. The number of hits for each country was summarized across pathogens creating an index variable reflecting the amount of *media attention* that IPs had received.

### Analyses

SPSS 22 statistics software was used to conduct the analyses. The public’s awareness of specific IPs in different countries was analyzed (standardized within country). Experiences of IPs, problem awareness, and policy acceptability were examined across countries (*n* = 3469). Multilevel models of awareness of IPs problem and policy acceptability were estimated using the linear mixed-effects model including level-1 and level-2 predictors (individual and country level, respectively). The cross-level interaction between media and awareness of IP problems was included in the model of policy acceptability assessing whether media moderate the influence of problem awareness (Table 4). The models were estimated using a reduced sample (*n* = 1983) because data for the number of IPs were not available for Bulgaria, Turkey, and Portugal, and “don’t know” responses were excluded. Because these analyses involved only six countries, we estimated parameters using a restricted maximum likelihood estimation (Hayes 2006), and a scaled identity covariance matrix was used for the random effects. Level-1 predictors were group mean centered and level-2 predictors were grand mean centered (Peugh 2010). The random effects in the unconditional models were not significant and the variation between countries was minor, explaining only 2.9 and 3.2% of the variance in awareness of IPs problem and policy acceptability, respectively (revealed by the Intra Class Correlations, ICC). However, to properly model all variables, we ran the multilevel models with variables entered as fixed effects. Results from the multilevel models include the unconditional model with no predictors, the model with only level-1 predictors and the full model for awareness of IPs problem and policy acceptability, respectively.

## Results

### Experience and public opinion

The public in the different countries had generally heard about at least one of the IPs listed, ranging from 43.6% in Norway to 84.9% in the UK (see Table 2). However, awareness of specific IPs varied greatly within and between countries. For example, while Dutch elm disease was the most well-known IP in the UK, Sweden, Norway, and Turkey, the pine wood nematode was more familiar to the public in Spain. Direct experience was not the main source of IP awareness, only 20.9% of the respondents had observed diseased trees in their local areas (answering 4 or 5 on the five-point scale). Moreover, less than a third (29.5%) reported personal experience of at least one of the eight IPs. Direct experience of IPs in their own garden or forest/woodland was very rare, instead most of the encounters had occurred in public parks and forests/woodlands. Almost half of the respondents (47.6%) had heard about at least one of the IPs in the media, mainly mass media.

**Fig. 1:**
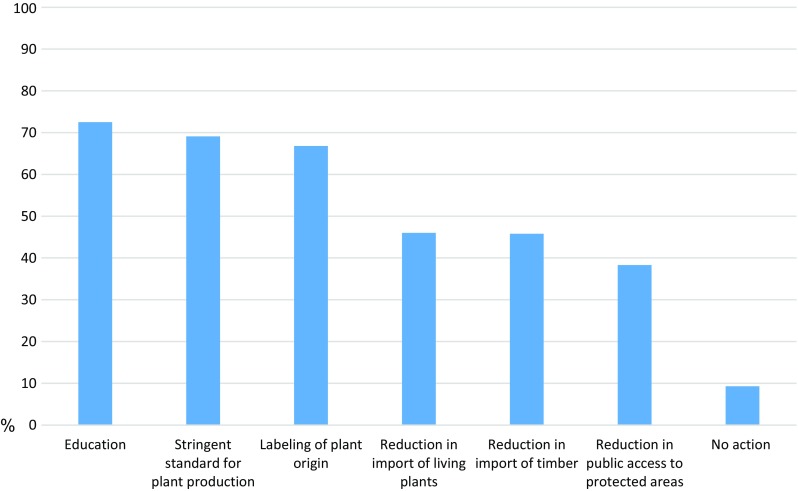
Overall policy acceptability in the nine European countries (share of respondents answering 4 or 5 on the five-point scale)

A larger share of the respondents considered tree disease to be a greater problem at a national level than at a local level (63.7% vs. 48.7% had answered 4 or 5 on the five-point scales). Respondents further believed that IPs have negative impacts on important forest values (*M* = 3.84, SD = 0.90). In general, the acceptability of the policies proposed was relatively high and the ‘no action’ option was the least preferred (Fig. 1). The respondents were mostly positive about the need for education; however, they also accepted more stringent health standards, and labeling of plant origin. Almost half of the respondents accepted a reduction of the import of living plants and timber products but only 38.3% were positive towards restricting public access to protected areas. Between-country variation in policy acceptability was minor (cf. the ICC). Education was among the most accepted policy measures in all countries, although more stringent health standards were equally accepted in the UK, France, Sweden, and Turkey. The ‘no action’ option was the least accepted measure in all countries.

### Multilevel modeling

Results from the multilevel models of awareness of IPs problem are displayed in Table 3 (including the unconditional model, the model with only level-1 predictors and the full model). Among the individual level predictors, less direct experience with IPs and more indirect experience, along with higher levels of awareness of tree disease problems, were associated with higher levels of awareness of IP problems. Even though the model with only level-1 predictors (individual level) displayed the best model fit (as suggested by the lower Akaike corrected and Bayesian values), level-2 predictors (country level) were significant suggesting that higher forest coverage of a country and more media coverage of IPs, but a lower forest production level, were related to a higher level of awareness of IP problems.

**Table 3:** Multilevel models of awareness of IP problems (unconditional model, level-1 predictors, full model) (coefficients and standard errors (SE))

	Unconditional model		Level-1 predictors		Full model	
	Parameters	SE	Parameters	SE	Parameters	SE
Fixed effects (regression coefficients)						
Level-1						
Intercept	3.792***	0.060	3.775***	0.058	3.791***	0.029
Gender (women)	–	–	0.098	0.061	0.098	0.061
Age	–	–	− 0.004	0.056	− 0.003	0.056
Education (natural sciences/agricultural)	–	–	− 0.052	0.056	− 0.053	0.056
Group (forest owner)	–	–	− 0.006	0.037	− 0.006	0.037
Place of residence (urban)	–	–	0.028	0.015	0.028	0.015
Direct experience of tree diseases	–	–	0.023	0.016	0.022	0.016
Direct experience of IPs	–	–	− 0.072*	0.033	− 0.072*	0.033
Indirect experience of IPs	–	–	0.113*	0.048	0.114*	0.048
Local tree disease problems	–	–	0.093***	0.018	0.093***	0.018
National tree disease problems	–	–	0.194***	0.032	0.194***	0.032
Level-2						
Share of forest	–	–	–	–	0.006*	0.003
Production	–	–	–	–	− 3.6 × 10^−6^*	1.4 × 10^−6^
Number of IPs	–	–	–	–	0.003	0.004
Media	–	–	–	–	7.3 × 10^−6^***	1.7 × 10^−6^
Random effects (variance components)						
Residual	0.765***	0.024	0.667***	0.021	0.667***	0.021
Intercept	0.023	0.016	0.023	0.016	0.022	0.035
Model summary						
Akaike corrected	5117.07		4887.03		4946.43	
Bayesian	5128.25		4898.20		4957.59	
Estimated parameters	3		13		17	

Dummy coding: gender: 0 = men, 1 = women, age: 0 = 18–64 years, 1 = 65 years or older, education: 0 = no, 1 = yes, place of residence: 0 = 10 000 residents or less, 1 = more than 10 000 residents, and forest owner: 0 = no, 1 = yes, direct/indirect experience: 0 = no, 1 = yes

*p < 0.05, ***p < 0.001

The multilevel models of policy acceptability are displayed in Table 4. Comparable to the model of awareness of IPs problem, the model with only level-1 predictors exhibited the best fit. Nonetheless, country-level variables and the cross-level interaction were significant. Policies to combat IPs were found to be more acceptable to women and older respondents than to their counterparts. Less direct experience, but more indirect experience, as well as higher levels of awareness of tree disease problems and IPs were related to higher levels of acceptability. A low forest production level and a high number of IPs in the country were furthermore linked to a higher level of policy acceptability. Media coverage did not significantly explain acceptability, although the significant interaction with awareness of IP problems suggests a stronger correlation between problem awareness and policy acceptability in countries with more media coverage of IPs. Overall, public opinion did not differ significantly between countries in Europe, and level-1 predictors (individual level), such as problem awareness and indirect experience, explained most of the variance in the public’s opinion of IPs. Nevertheless, level-2 predictors (country level), such as media attention, provide insights into how external conditions can influence public opinion.

**Table 4:** Multilevel models of policy acceptability (unconditional model, level-1 predictors, full model) (coefficients and standard errors (SE))

	Unconditional model		Level-1 predictors		Full model	
	Parameters	SE	Parameters	SE	Parameters	SE
Fixed effects (regression coefficients)						
Level-1						
Intercept	3.756***	0.051	3.747***	0.051	3.767***	0.029
Gender (women)	–	–	0.041*	0.018	0.040*	0.017
Age	–	–	0.100***	0.011	0.098***	0.011
Education (natural sciences/agricultural)	–	–	− 0.051	0.067	− 0.055	0.066
Group (forest owner)	–	–	− 0.021	0.039	− 0.019	0.039
Place of residence (urban)	–	–	0.012	0.026	0.010	0.026
Direct experience of tree diseases	–	–	− 0.008	0.010	− 0.008	0.010
Direct experience of IPs	–	–	− 0.067*	0.028	− 0.069*	0.029
Indirect experience of IPs	–	–	0.143***	0.019	0.138***	0.017
Local tree disease problems	–	–	0.044***	0.010	0.044***	0.010
National tree disease problems	–	–	0.065***	0.015	0.063***	0.014
IP problems	–	–	0.233***	0.025	0.239***	0.015
Level-2						
Share of forest	–	–	–	–	0.001	0.003
Production	–	–	–	–	− 3.1 × 10^−6^*	1.4 × 10^−6^
Number of IPs	–	–	–	–	0.016***	0.003
Media	–	–	–	–	2.7 × 10^−6^	1.7 × 10^−6^
L2–L1 interaction						
Media × IP problems	–	–	–	–	2.1 × 10^−6^***	2.7 × 10^−7^
Random effects (variance components)						
Residual	0.390***	0.012	0.311***	0.010	0.310***	0.010
Intercept	0.017	0.012	0.018	0.012	0.023	0.033
Model summary						
Akaike corrected	3786.27		3389.86		3467.47	
Bayesian	3797.45		3401.03		3478.63	
Estimated parameters	3		14		19	

Dummy coding: gender: 0 = men, 1 = women, age: 0 = 18–64 years, 1 = 65 years or older, education: 0 = no, 1 = yes, place of residence: 0 = 10 000 residents or less, 1 = more than 10 000 residents, and forest owner: 0 = no, 1 = yes, direct/indirect experience: 0 = no, 1 = yes

*p < 0.05, ***p < 0.001

## Discussion

The threat from IPs and history of former disease outbreaks by IPs differ between European countries because climate conditions and the forests in Europe are diverse (Santini et al. 2013). In addition, not only the amount of forested areas, forestry’s contribution to national economy and recreational use of forests, but also the volume of trade of different plants and wood products vary considerably. Variation in public awareness of IPs among the surveyed countries is therefore reasonable; however, the reasons behind such variation can be hard to infer. For example, the high level of public awareness of the Dutch elm disease in the UK may be considered against the background of the once high importance of elms in the UK and outbreaks of the disease coupled with strong media attention in the country (Urquhart et al. 2017). Similarly, public awareness of pine wood nematode in the Iberian Peninsula in contrast to other countries could be explained by the fact that only Spain and Portugal are affected so far in Europe. However, the level of public awareness in Portugal was lower than expected. More generally, public awareness for specific IPs was unexpectedly high in some countries, for example, Pine pitch canker in France, which is not yet present contrary to Spain. In contrast, awareness of alder *Phytophthora* was very low in all countries. The awareness of ash dieback in the UK was furthermore lower than expected (especially compared to Dutch elm disease) when considering a recent invasion with extensive press coverage and a strong involvement of political powers (Woodward and Boa 2013).

Despite large variations in the awareness of specific IPs, the public in different European countries displayed a fairly coherent view both in terms of awareness of IP problems and policy acceptability (as suggested by the low level of between-country variance). Although IPs were generally believed to have a negative impact on important forest values, the issue of tree diseases in general was considered a more distant rather than a local threat, comparable to how the public perceives climate change (McDonald et al. 2015). Low awareness of specific IPs, but a larger awareness of the problem in general, is in line with previous research (Urquhart et al. 2017). This result is encouraging since awareness of every single specific IP is most likely not needed in order to raise public support for collective actions.

Given the increasing rate of pathogen invasions (Santini et al. 2013), it is furthermore promising that the public’s acceptance for policy actions was high. The highest acceptance was found for the informational measures, which suggests that people believe there is a need to increase awareness of IPs and that they would like to, for instance, make informed decisions as plant consumers. Strategic provisioning of information that explains how people contribute to problems associated with IPs and what they can do to alleviate the problem (e.g., at the entrance of recreational areas and plant stores) may be one way of making this issue salient to the public. In addition, the public’s acceptance of more stringent measures for plant production and import was also reasonably high. Thus, suggesting that also political actions with potential consequences to the availability of plants for horticultural use might be accepted. The public was less positive towards restricting public access to protected areas. This may either originate from the disbelief that recreational activities contribute much to the spread of IPs, or indicate that the public is less willing to accept policies restricting their personal freedom, comparable to results in other policy domains (e.g., Jakobsson et al. 2000). Importantly, the survey asked about policy measures without providing any details on the implications or explicitly stating who would be responsible for the implementation costs. Public acceptance would likely be lower if the public would pay for the measures compared to if the industry would bear the cost for example. Since behaviors are not always fully aligned with attitudes (Eagly and Chaiken 1993), high public acceptance does not necessarily suggest that people will themselves take action against IPs but, rather, that political action is considered legitimate.

By revealing how external conditions and individual level variables are related to problem awareness and policy acceptability, our study sheds light on how the public’s opinion about IPs is formed. The analyses of external conditions suggest that problems with IPs are emphasized more strongly in countries with larger forest cover and more media coverage of IPs. In addition, a high number of IPs in a country seem to stimulate support for policy. Such external conditions may essentially reflect an increased attention to IPs in the country, which then plays a role in shaping public opinion. Remarkably, awareness of IP problems and policy acceptability were lower in countries with higher levels of forest production, potentially indicating an uncoupling of issues (e.g., IPs may not be given much attention in debates on forest production). The study revealed that external conditions mattered for public opinion on IPs, but the robust effects of experiences and cognitions on awareness of IPs problem and policy acceptability emphasize the importance of the individual level (cf. Qin and Flint 2010).

In general, socio-demographics played a lesser role for public perceptions of IPs than the psychological variables. Nevertheless, women and older respondents displayed higher policy acceptability than their counterparts, and comparable results have been found for willingness to adopt biosecurity behaviors (Urquhart et al. 2017). In contrast, the support for the management of IPs has in other studies been found to be stronger among men than women, while the influence of age depended on the management method (Fuller et al. 2016; Jepson and Arakelyan 2017). Contrary to the expectation that directly experiencing a risk should make people more willing to respond (Reser et al. 2014), this study revealed that direct experience of IPs was negatively correlated with awareness of IP problems and support for collective actions. Personal experiences of pathogen outbreaks may be severe, involving for example a sense of personal loss and damage to economic, aesthetic, and wildlife values (Porth et al. 2015). However, if a risk experience is not severe, personal experience may reduce rather than increase willingness to act (Weinstein 1989). In contrast, more attention in the media, indirect experience, and awareness of the problem increased policy acceptability. Furthermore, media attention strengthened the relationship between problem awareness and policy acceptability. Media may thus not only amplify risk signals during a disease outbreak (Fellenor et al. 2017), but through continued attention influence public opinion. Even though a rough indicator of media attention (based on google search) was used in the present study, the importance of media was confirmed across several IPs; thus, our results can complement case studies of how the media influence public perceptions of particular IPs (Tomlinson 2016; Fellenor et al. 2017).

When interpreting the results there are limitations to consider. While not randomly selected, quotas were used to select the samples (cf. Fuller et al. 2016; Urquhart et al. 2017), and in seven of the nine countries, the samples were representative regarding age and gender. Furthermore, in the multilevel models, the countries with deviating socio-demographic distributions (i.e., Bulgaria and Turkey) were not included and the influence of socio-demographics was controlled for. Answers to surveys are known to be influenced by different biases (e.g., response bias), and these may be particularly challenging in cross-cultural research. Although we paid very careful attention to the formulation of questions, the naming of diseases in the various languages may have still introduced some biases. Nevertheless, to the extent that we can compare, results were overall consistent with previous studies on public awareness of IPs (Urquhart et al. 2017). The data are furthermore correlational which prevent casual claims. While limitations should be taken into account, the analyses were theoretically justified, and the results from the survey constitute a broad empirical base. Consequently, this study can provide important insights into public awareness of IPs and policy acceptability in this domain.

## Conclusions

A cross-country survey of the public in nine European countries revealed considerable variation in the awareness of specific IPs. Nevertheless, there were only minor country differences in the public’s awareness of the problems associated with IPs and acceptance of policies aiming to combat IPs on a European scale. In addition to informational measures including labeling of plant origin, the public showed rather high acceptance of stringent measures for plant production. However, lower acceptance was observed for restricting public access to protected areas. Results further suggest that learning about IPs through the media and recognizing the problems associated with IPs increase policy acceptability. Whereas direct experience has been examined in relation to climate change (Demski et al. 2017), the present study emphasizes the role of indirect experience in support for collective actions in relation to gradually progressing and highly complex environmental risks, such as IPs.
